# Supplementary material for: Single-component multilayered self-assembling protein nanoparticles presenting glycan-trimmed uncleaved prefusion optimized envelope trimers as HIV-1 vaccine candidates
Source: Nat Commun. 2023 Apr 8;14:1985. doi: 10.1038/s41467-023-37742-z (PMC10082823; doi:10.1038/s41467-023-37742-z)
Supplement: Supplementary file 3 — Reporting Summary [file 41467_2023_37742_MOESM3_ESM.pdf]

nature portfolio

Corresponding author(s): Jiang Zhu

Last updated by author(s): Mar 25, 2023

Reporting Summary

Nature Portfolio wishes to improve the reproducibility of the work that we publish. This form provides structure for consistency and transparency in reporting. For further information on Nature Portfolio policies, see our Editorial Policies and the Editorial Policy Checklist.

Statistics

For all statistical analyses, confirm that the following items are present in the figure legend, table legend, main text, or Methods section.

n/a	Confirmed	
☐	☒	The exact sample size (n) for each experimental group/condition, given as a discrete number and unit of measurement
☐	☒	A statement on whether measurements were taken from distinct samples or whether the same sample was measured repeatedly
☐	☒	The statistical test(s) used AND whether they are one- or two-sided<br>*Only common tests should be described solely by name; describe more complex techniques in the Methods section.*
☐	☒	A description of all covariates tested
☐	☒	A description of any assumptions or corrections, such as tests of normality and adjustment for multiple comparisons
☐	☒	A full description of the statistical parameters including central tendency (e.g. means) or other basic estimates (e.g. regression coefficient) AND variation (e.g. standard deviation) or associated estimates of uncertainty (e.g. confidence intervals)
☐	☒	For null hypothesis testing, the test statistic (e.g. F, t, r) with confidence intervals, effect sizes, degrees of freedom and P value noted<br>*Give P values as exact values whenever suitable.*
☒	☐	For Bayesian analysis, information on the choice of priors and Markov chain Monte Carlo settings
☒	☐	For hierarchical and complex designs, identification of the appropriate level for tests and full reporting of outcomes
☒	☐	Estimates of effect sizes (e.g. Cohen's d, Pearson's r), indicating how they were calculated

Our web collection on statistics for biologists contains articles on many of the points above.

Software and code

Policy information about availability of computer code

Data collection

SEC data were collected using the Unicorn 7.5 software (GE healthcare). SDS-PAGE and BN-PAGE images were collected using the Image Lab v6.0 software. BLI (Octet) data were collected by the Data acquisition 8.2 (FORTEBIO). DSC data were collected by the MicroCal PEAQ-DSC software v1.52 (Malvern Panalytical). DLS data were collected by a Zetasizer Ultra instrument (Malvern). ELISA data were collected using the PerkinElmer 2030 v4.0 software (PerkinElmer). Neutralization data were collected using the Gen5 software (Biotek). TEM images of negative stained BG505 UFO trimer presenting nanoparticle and lymph node samples were collected at 80 kV with a Talos L120C transmission electron microscope (Thermo Fisher), and images were acquired with a CETA 16M CMOS camera. Cryo-EM data were collected from two microscopes, a Talos Arctica TEM (Thermo Fisher Scientific) operating at 200 kV, and an FEI Titan Krios TEM (Thermo Fisher Scientific) at 300 kV. Both microscopes were equipped with the Gatan K2 Summit direct electron detector camera and sample autoloader. The Leginon software (Version beta) suite was used for automated data acquisition. Glycopeptide fragmentation data were extracted from the raw file using Byonic (Version 3.5) and Byologic software (Version 3.5; Protein Metrics). Immunohistology data were collected using an Olympus VS-120 slide scanner with a Hamamatsu ORCA-R2 C10600 digital camera. Flow cytometry data were collected using a 5-laser AZE5 flow cytometer (Yeti, Bio-Rad) with Everest software.

Data analysis

GraphPad Prism 9.3.1 was used to analyze and plot DSC, DLS, ELISA and neutralization data. BLI (Octet) data were analyzed by Data analysis 8.2 (FORTEBIO). The structural analysis of EM images was performed using CryoSPARC 2 software on the Scripps Garibaldi cluster. Localized reconstruction method was used to analyze images of the BG505 UFO trimer presenting nanoparticles obtained from Cryo-EM. Global glycan analysis were processed using Empower 3 software. Immunohistological images were analyzed using ImageJ software. Flow cytometry data were analyzed using FlowJo 10 software.

For manuscripts utilizing custom algorithms or software that are central to the research but not yet described in published literature, software must be made available to editors and reviewers. We strongly encourage code deposition in a community repository (e.g. GitHub). See the Nature Portfolio guidelines for submitting code & software for further information.

Data

Policy information about availability of data

All manuscripts must include a data availability statement. This statement should provide the following information, where applicable:

- Accession codes, unique identifiers, or web links for publicly available datasets
- A description of any restrictions on data availability
- For clinical datasets or third party data, please ensure that the statement adheres to our policy

The EM data generated in this study have been deposited in the Electron Microscopy Data Bank (EMDB, https://www.ebi.ac.uk/emdb/), under accession codes EMD-28540 [https://www.ebi.ac.uk/emdb/EMD-28540], EMD-28541 [https://www.ebi.ac.uk/emdb/EMD-28541], EMD-28542 [https://www.ebi.ac.uk/emdb/EMD-28542], EMD-28543 [https://www.ebi.ac.uk/emdb/EMD-28543], and EMD-28555 [https://www.ebi.ac.uk/emdb/EMD-28555]. The structural data have been deposited in the Protein Data Bank (PDB, https://www.rcsb.org/), under accession code 8EQN [www.rcsb.org/structure/8EQN]. RAW files associated with the site-specific glycan analysis can be accessed at massive.ucsd.edu under doi:10.25345/C55D8NQ7W. The authors declare that the data supporting the findings of this study are available within the article and its Supplementary Information files. Source data are provided with this paper.

Human research participants

Policy information about studies involving human research participants and Sex and Gender in Research.

Reporting on sex and gender	N/A
Population characteristics	N/A
Recruitment	N/A
Ethics oversight	N/A

Note that full information on the approval of the study protocol must also be provided in the manuscript.

Field-specific reporting

Please select the one below that is the best fit for your research. If you are not sure, read the appropriate sections before making your selection.

☒ Life sciences ☐ Behavioural & social sciences ☐ Ecological, evolutionary & environmental sciences

For a reference copy of the document with all sections, see nature.com/documents/nr-reporting-summary-flat.pdf

Life sciences study design

All studies must disclose on these points even when the disclosure is negative.

Sample size	The numbers used in the current study are rather standard for the field and have been used in our previous studies (e.g. groups of 3-10 mice and 4 rabbits used in He et al., Sci Adv, 4:eaau6769, 2018; He et al., Sci Adv, 6:eaaz6225, 2021; Zhang et al., Sci Adv, 7: eabj3107, 2021; He et al., Nat Comm, 12:2633, 2021). Here, 3-10 mice per group, 6 rabbit per group, and 4-6 nonhuman primates per group were used for immunogenicity and mechanistic studies. The sample size provides statistical significance in the key experiments.
Data exclusions	Seven mice were excluded from the neutralization assays of mouse IgG from week 11, as they died before reaching the final time point.
Replication	Antibody binding to UFO trimer and timer presenting nanoparticles was performed in singlet using BLI (Octet), with a separate Octet binding experiment performed in duplicate for the E2p nanoparticle to assess the variability between technical replicates. Neutralization assays using purified mouse IgG or sera from mouse, rabbit and nonhuman primates were performed with duplicates, except for some mice in the MLV and SF162 assays and all mice in the 12-virus assays due to the limited sample availability.
Randomization	No randomization was performed for mice and rabbits, because the animals in this study were homogeneous in sex, age, and weight. Six-to-eight-week-old female BALB/c wild type mice were purchased from The Jackson Laboratory. Three-to-four-month-old female New Zealand White rabbits of were performed through subcontract to Covance (Denver, PA) and ProSci (San Diego, CA). The Research-naive adult rhesus macaques of Indian origin were sourced from the Southwest National Primate Research Center (SNPRC) and Tulane National Primate Research Center (TNPRC). These macaques were randomly assigned to the study groups, which were balanced for age, weight, and gender.
Blinding	The animals in this study (mice, rabbits and rhesus macaques) were homogeneous in sex, age, and weight. Blinding is not relevant in this study. Animal samples were collected and analyzed using standardized protocols and assays with proper controls. There is no subjective assessment of the animals and the collected animal samples.

Reporting for specific materials, systems and methods

We require information from authors about some types of materials, experimental systems and methods used in many studies. Here, indicate whether each material, system or method listed is relevant to your study. If you are not sure if a list item applies to your research, read the appropriate section before selecting a response.

Materials & experimental systems

n/a	Involved in the study
☐	☒ Antibodies
☐	☒ Eukaryotic cell lines
☒	☐ Palaeontology and archaeology
☐	☒ Animals and other organisms
☒	☐ Clinical data
☒	☐ Dual use research of concern

Methods

n/a	Involved in the study
☒	☐ ChIP-seq
☐	☒ Flow cytometry
☒	☐ MRI-based neuroimaging

Antibodies

Antibodies used

Anti-human IgG (Jackson ImmunoResearch #109-035-008), and anti-mouse IgG (Jackson ImmunoResearch #115-036-008) conjugated to horseradish peroxidase (HRP) were used in ELISA binding assays.

A panel of bNAbs and non-NAbs was utilized to characterize the antigenicity of various native-like trimers and gp140 nanoparticles. Antibodies were requested from the NIH AIDS Reagent Program (https://www.aidsreagent.org/) except for 438-B11, PGT151, M4H2K1, SF12, and RM20A3, which were produced in-house together with the fragment antigen-binding (Fab) regions of PGT128 and VRC01.

For immunohistological study, lymph node sections were stained with bNAbs VRC01, PGT124, and PGDM1400 (1:50), and biotinylated goat anti-human secondary antibody (Abcam, catalog no. ab7152, 1:300), followed by streptavidin-horseradish peroxidase (HRP) reagent (Vectastain Elite ABC-HRP Kit, Vector, catalog no. PK-6100) and diaminobenzidine (DAB) (ImmPACT DAB, Vector, catalog no. SK-4105). FDCs were labeled using anti-CD21 primary antibody (Abcam, catalog no. ab75985, 1:1800), followed by anti-rabbit secondary antibody conjugated with Alexa Fluor 555 (Thermo Fisher, catalog no. A21428, 1:200). B cells were labeled using anti-B220 antibody (eBioscience, catalog no. 14-0452-82, 1:100) followed by anti-rat secondary antibody conjugated with Alexa Fluor 647 (Thermo Fisher, catalog no. A21247, 1:200). Subcapsular sinus macrophages were labeled using anti-sialoadhesin (CD169) antibody (Abcam, catalog no. ab53443, 1:600) followed by anti-rat secondary antibody conjugated with Alexa Fluor 488 (Abcam, catalog no. ab150165, 1:200). HIV-1 vaccine-induced GCs were studied using immunostaining, GC B cells stained using rat anti-GL7 antibody (FITC; BioLegend, catalog no. 144604, 1:250), Tfh cells stained using anti-CD4 antibody (BioLegend, catalog no. 100402, 1:100) followed by anti-rat secondary antibody conjugated with Alexa Fluor 488 (Abcam, catalog no. ab150165, 1:1000), GC cells stained using Bcl6 antibody (Abcam, catalog no. ab220092, 1:300) followed by anti-rabbit secondary antibody conjugated with Alexa Fluor 555 (Thermo Fisher, catalog no. A21428, 1:1000). Nuclei were labeled using 4′,6-diamidino-2-phenylindole (DAPI) (Sigma-Aldrich, catalog no. D9542, 100 ng/ml)

For flow cytometry study, Anti-CD16/32 antibody (BioLegend, catalog no. 101302, 1:50) was added to block the nonspecific binding of Fc receptors. lymph node samples were stained with cocktail antibodies, which included the Zombie NIR live/dead stain (BioLegend, catalog no. 423106, 1:100), Brilliant Violet 510 anti-mouse/human CD45R/B220 antibody (BioLegend, catalog no. 103247, 1:300), FITC anti-mouse CD3 antibody (BioLegend, catalog no. 100204, 1:300), Alexa Fluor 700 anti-mouse CD4 antibody (BioLegend, catalog no. 100536, 1:300), PE anti-mouse/human GL7 antibody (BioLegend, catalog no. 144608, 1:500), Brilliant Violet 605 anti-mouse CD95 (Fas) antibody (BioLegend, catalog no. 152612, 1:500), Brilliant Violet 421 anti-mouse CD185 (CXCR5) antibody (BioLegend, catalog no. 145511, 1:500), and PE/Cyanine7 anti-mouse CD279 (PD-1) antibody (BioLegend, catalog no. 135216, 1:500).

Validation

All the commercially available antibodies have been validated by their manufacturers, Jackson ImmunoResearch, BD Biosciences, Abcam, Thermo Fisher, Sigma-Aldrich and BioLegend. All antibodies were validated by the manufacturers via western blot analysis and immunofluorescence as documented in the manufacturers' websites.

The ELISA and BLI binding assays and the neutralization assays performed in this study validated the function of these antibodies. The immunohistological analysis and flow cytometry study performed in this study also validated the use of these antibodies.

Eukaryotic cell lines

Policy information about cell lines and Sex and Gender in Research

Cell line source(s)

ExpiCHO cells (Thermo Fisher, catalog no. A29133)
CHO-K1 host cell line (ATCC, no. CCL-61)
HEK293T cells (ATCC, no. CRL-3216)
TZM-bl cells (NIH AIDS Reagent program)

Authentication

These commonly used cell lines were obtained from and authenticated by the vendors and government agencies. Validation reports can be found on their websites. No authentication was performed after purchase.

March 2021

Mycoplasma contamination	The cell lines were not contaminated by mycoplasma as determined by using the Lonza Mycoplasma Detection Kit.
Commonly misidentified lines (See ICLAC register)	No commonly misidentified cell lines were used in the study

Animals and other research organisms

Policy information about studies involving animals; ARRIVE guidelines recommended for reporting animal research, and Sex and Gender in Research

Laboratory animals	Six-to-eight-week-old female BALB/c wild type mice were purchased from The Jackson Laboratory. Mice were housed in a controlled environment at approximately 20 °C, 50% humidity and 12 hour light-dark cycles. Three-to-four-month-old female New Zealand White rabbits of were performed by a subcontract at Covance (Denver, PA) and ProSci (San Diego, CA). Research-naive female and male adult rhesus macaques of Indian origin (age 4.0-11.5 years old) were sourced from the Southwest National Primate Research Center (SNPRC) and Tulane National Primate Research Center (TNPRC).
Wild animals	No wild animals were used in the study.
Reporting on sex	All BALB/c mice and New Zealand White rabbits used in this study are females. Rhesus macaques include both females and males.
Field-collected samples	No field collected samples were used in the study.
Ethics oversight	The mouse immunization was performed following the Institutional Animal Care and Use Committee (IACUC) protocol approved by The Scripps Research Institute. The rabbit immunization was performed through subcontract to Covance and ProSci. The IACUC guidelines were followed for the animal subjects tested in the immunization studies. Rhesus macaques were sourced from the Southwest National Primate Research Center (SNPRC) and Tulane National Primate Research Center (TNPRC). All experimental procedures were performed at SNPRC in San Antonio, TX, USA according to the guidelines of the Association for Assessment and Accreditation of Laboratory Animal Care (AAALAC) standards. These macaque experiments were carried out in compliance with all pertinent US National Institutes of Health (NIH) regulations and were approved by the IACUC of SNPRC.

Note that full information on the approval of the study protocol must also be provided in the manuscript.

Flow Cytometry

Plots

Confirm that:

☒ The axis labels state the marker and fluorochrome used (e.g. CD4-FITC).

☒ The axis scales are clearly visible. Include numbers along axes only for bottom left plot of group (a 'group' is an analysis of identical markers).

☒ All plots are contour plots with outliers or pseudocolor plots.

☒ A numerical value for number of cells or percentage (with statistics) is provided.

Methodology

Sample preparation	Mice were euthanized at 2, 5, and 8 weeks after a single-dose injection and at 2 and 5 weeks after the boost, which occurred at 3 weeks after the first dose (4 footpads, 10 µg/footpad). Fresh axillary, brachial, and popliteal sentinel lymph nodes were isolated. After mechanically disaggregating the lymph node tissues, samples were merged in enzyme digestion solution in an Eppendorf tube containing 958 µl of Hanks' balanced salt solution (HBSS) buffer (Thermo Fisher Scientific, catalog no. 14185052), 40 µl of 10 mg/ml collagenase IV (Sigma-Aldrich, catalog no. C5138), and 2 µl of 10 mg/ml DNase (Roche, catalog no. 10104159001). Lymph node tissues were incubated at 37°C for 30 min and filtered through a 70 µm cell strainer. Samples were spun down at 400 g for 10 min to isolate cell pellets, which were resuspended in HBSS blocking buffer with 0.5% (w/v) bovine serum albumin and 2 mM EDTA. Anti-CD16/32 antibody as added to block the nonspecific binding of Fc receptors, while the sample solution was kept on ice for 30 min. Samples were then transferred to 96-well microplates with pre-prepared cocktail antibodies. The cell samples mixed with antibody cocktail were placed on ice for 30 min and centrifuged to remove excess antibody. After washing with the HBSS blocking solution, cells were fixed with 1.6% paraformaldehyde (Thermo Fisher Scientific, catalog no. 28906) in HBSS on ice for 30 min. The samples were then placed in HBSS blocking solution at 4°C. Sample events were acquired by a 5-laser AZE5 flow cytometer (Yeti, Bio-Rad) with Everest software at the Core Facility of The Scripps Research Institute. The data were analyzed using FlowJo 10 software.
Instrument	5-laser AZE5 flow cytometer (Yeti, Bio-Rad).
Software	Everest software, FlowJo 10 software, GraphPad Prism 9.3.1.
Cell population abundance	B cells, T Cells and their subtypes.
Gating strategy	Germinal center B cells and Tfh cells were gated based on the GL7+B220+CD95+ and CD3+CD4+CXCR5+PD-1+, respectively.

☒ Tick this box to confirm that a figure exemplifying the gating strategy is provided in the Supplementary Information.
